# Subcutaneous Panniculitis-Like T-Cell Lymphoma Presenting as a Local Inflammation of a Thigh in an 8-Month-Old Child

**DOI:** 10.1055/s-0037-1607036

**Published:** 2017-10-28

**Authors:** Kaja Gizewska-Kacprzak, Katarzyna Karpinska-Kaczmarczyk, Tomasz Ociepa

**Affiliations:** 1 Department of Pediatric and Oncological Surgery, Pomorski Uniwersytet Medyczny w Szczecinie, Szczecin, Poland; 2 Department of Pathology, Pomorski Uniwersytet Medyczny w Szczecinie, Szczecin, Poland; 3 Department of Pediatrics, Hematology and Oncology, Pomorski Uniwersytet Medyczny w Szczecinie, Szczecin, Poland

**Keywords:** cutaneous T-cell lymphoma, child, subcutaneous panniculitis-like T-cell lymphoma, immunohistochemistry

## Abstract

During infancy, skin inflammation is usually treated in basic pediatric care. In this study, we present a case of an 8-month-old girl with a 2-month history of an inflammation of the thigh treated locally by ointments and oral antibiotics in basic and dermatological care. The patient had a history of fever, sweating, and failure to thrive. The lactate dehydrogenase was elevated up to 869 U/L with low C-reactive protein (1.04 mg/L). Magnetic resonance imaging of the thigh reassured the diagnosis of local inflammation. Intravenous antibiotic caused mild local improvement, but the episodes of high fever sustained. The patient was transferred to our pediatric surgery department for treatment and surgical biopsy of the lesion. Histopathological examination confirmed a subcutaneous panniculitis-like T-cell lymphoma, which is a rare cytotoxic T-cell lymphoma representing less than 1% of non-Hodgkin lymphomas, uncommon in children. The patient was introduced to a chemotherapy protocol EURO-LB 2002 with good response. In a skin lesion that is associated with systemic symptoms and responding untypically to antibiotic treatment malignancy should be considered and biopsy not be postponed.

## Introduction

Almost 30% of pediatric primary care visits regard skin-related symptoms. 1 From the neonatal period throughout infancy, most of the skin lesions are benign, and self-limiting. 2 Differential diagnosis of local skin inflammation usually includes trauma, insect bites, atopic dermatitis, allergies, or inappropriate hygiene. 3 We report on an infant with a cellulitis of the thigh which turned out to be a subcutaneous T-cell lymphoma.

## Case Report

**Fig. 1:**
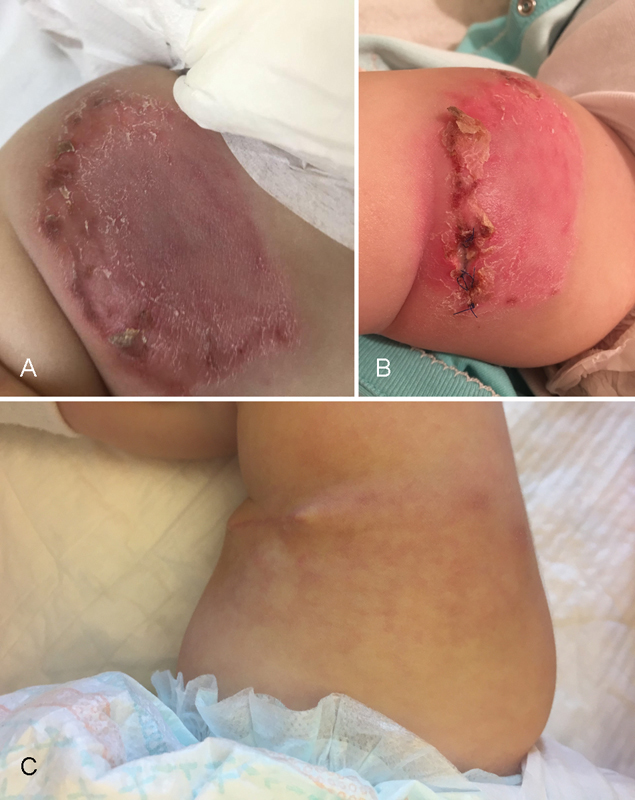
( **A** ) The clinical picture of the right thigh after 2-months of conservative treatment; ( **B** ) the lesion of the thigh after surgical biopsy (visible skin sutures) that confirmed the diagnosis; ( **C** ) the same skin surface after 4 months of chemotherapy.

An 8-month-old girl with a 2-month history of superficial cellulitis of the anterior-medial right thigh treated conservatively with ointments and local/oral antibiotics in basic and dermatological care with no clinical improvement. Clinically, a large (10 × 5 cm) single, rectangular, flat, stiff infiltration with redness and peeling of the skin was observed ( Fig. 1A ). The patient had a history of fever, sweating, and failure to thrive. Shortly before hospitalization at our surgical unit in the pediatric oncology department, laboratory tests showed elevated lactate dehydrogenase up to 869 U/L, aspartate transaminase up to 153 U/L, alanine transaminase up to 113 U/L, and low C-reactive protein (1.04 mg/L). However, a magnetic resonance imaging (MRI) of a thigh confirmed the diagnosis of a local inflammation with mildly enlarged lymph node of the groin. The intravenous antibiotic was introduced causing mild local improvement, but the episodes of high fever sustained. The patient was transferred to our pediatric surgery department for treatment and surgical biopsy of the lesion ( Fig. 1B ).

**Fig. 2:**
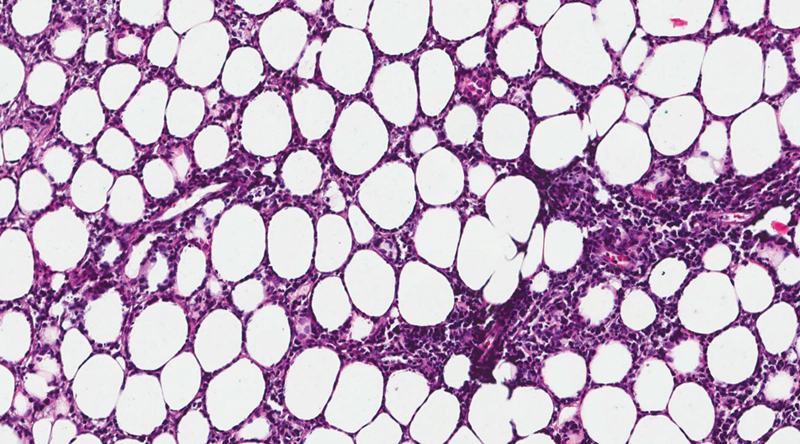
Hematoxylin and eosin staining (extension ×200). Skin biopsy showing subcutaneous tissue with neoplastic infiltrate. The neoplastic cells range in size and have irregular, hyperchromatic nuclei. The characteristic feature is the rimming of the neoplastic cells surrounding individual fat cells.

Histopathological examination showed a massive T-cell infiltration (cluster of differentiation [CD]) CD3+, CD8+, CD5+, CD7+, CD4+ with sporadic CD99+, CD34+, TdT-, CD10-, CD56- cells with numerous histiocytes, plasmatic cells and neutrophils. A very high proliferative index was noticed (Ki-67+ > 70% of cells). A subcutaneous panniculitis-like T-cell lymphoma (SPTCL) was diagnosed. Characteristic rimming of individual fat cells by tumor cells with immunohistochemical staining specific for SPTCL was visualized in our patient's specimen ( Fig. 2 ).

The patient was transferred to the pediatric hematology department. Further staging included a whole-body computed tomography (CT) scan with contrast enhancement (as a whole-body MRI was unavailable and patient's general status was worsening). The patient was introduced to a special chemotherapy protocol EURO-LB 2002 (prednisone, 6-mercaptopurine, and methotrexate) mildly adjusted to young age, with a good response. During the first weeks of treatment, there was a *Pseudomonas aeruginosa* infection of the wound after the surgical biopsy which was treated with targeted antibiotic therapy and later underwent surgical debridement with good wound healing ( Fig. 1C ). A control MRI of the thigh showed a significant improvement with reduction of the lesion without enlargement of the surrounding lymph nodes.

## Discussion

The SPTCL is a rare cytotoxic T-cell lymphoma representing less than 1% of non-Hodgkin lymphomas. 4 It predominantly affects young adults and is uncommon in children. 5 SPTCL preferentially infiltrates subcutaneous tissue. As observed in our case, the most common sites of localization are the extremities and trunk. Systemic symptoms are observed in 50% of patients. 5 Our patient experienced intensive sweating and fever episodes for a significant time period while treatment was only local without any further diagnostics. Definitive diagnosis is based on a complex histopathological examination. 6 Nevertheless, it is crucial to combine the biopsy with information about clinical symptoms to guide the pathologist in search of the accurate interpretation. In the presented diagnostic process, in the communication between surgeon and pathologist information about general symptoms as well as elevated parameters were very useful and led to the final diagnosis.

Based on published case series, the morbidity, and mortality related to the development of hemophagocytic syndrome that aggravates the prognosis, which optimistically was not present in our patient. 7 Further molecular diagnostic tests determine T-cell receptor phenotype. The World Health Organization (WHO) and European Organization for Research and Treatment of Cancer (EORTC) in 2008 distinguished SPTCL with alpha beta T cells from primary cutaneous gamma delta lymphoma (PCGD-TCL). 6 Immunophenotypic differences include CD56 positivity with lack of CD4 and CD8 in PCGD-TCL. Such a division correlates with differences in prognosis, with much better survival rates in SPTCL. 8

The rarity of SPTCL resulted in a wide range of treatment strategies: from no treatment, steroids, immunosuppressive therapy to multiagent chemotherapy. 8 In the presented case a chemotherapy protocol was introduced due to extensive general symptoms and progression of the local state. If available, a whole-body MRI should be considered in the staging process rather than a whole-body CT due to the high levels of radiation connected with the latter.

In a skin lesion that is associated with systemic symptoms and responding untypically to antibiotic treatment malignancy should be considered and biopsy not be postponed.

## New Insights and the Importance for the Pediatric Surgeon

Our case of an extremely rare subcutaneous panniculitis-like T-cell lymphoma is a guideline for a pediatric surgeon that in a skin lesion associated with systemic symptoms and responding untypically to antibiotic treatment malignancy should be considered and biopsy not be postponed.
